# ENHANCED RECOVERY (ERAS) AFTER LIVER SURGERY:COMPARATIVE STUDY IN A BRAZILIAN TERCIARY CENTER

**DOI:** 10.1590/0102-672020180001e1424

**Published:** 2019-02-07

**Authors:** Uirá Fernandes TEIXEIRA, Marcos Bertozzi GOLDONI, Fábio Luiz WAECHTER, José Artur SAMPAIO, Florentino Fernandes MENDES, Paulo Roberto Ott FONTES

**Affiliations:** 1 Hepatobiliary and Pancreatic Surgical Division; 2 Department of Anesthesiology, Federal University of Health Sciences of Porto Alegre / Santa Casa de Misericórdia de Porto Alegre, Porto Alegre, RS, Brazil

**Keywords:** Hepatectom, Length of Sta, Recovery of Functio, Postoperative Care, Hepatectomi, Tempo de Internaçã, Recuperação da função fisiológic, Cuidados pós-operatórios

## Abstract

**Background:**

After the publication of the first recommendations of ERAS Society regarding colonic surgery, the proposal of surgical stress reduction, maintenance of physiological functions and optimized recovery was expanded to other surgical specialties, with minimal variations.

**Aim:**

To analyze the implementation of ERAS protocols for liver surgery in a tertiary center.

**Methods:**

Fifty patients that underwent elective hepatic surgery were retrospectively evaluated, using medical records data, from June 2014 to August 2016. After September 2016, 35 patients were prospectively evaluated and managed in accordance with ERAS protocol.

**Results:**

There was no difference in age, type of hepatectomy, laparoscopic surgery and postoperative complications between the groups. In ERAS group, it was observed a reduction in preoperative fasting and in the length of hospital stay by two days (p< 0.001). Carbohydrate loading, j-shaped incision, early oral feeding, postoperative prevention of nausea and vomiting and early mobilization were also significantly related to ERAS group. Oral bowel preparation, pre-anesthetic medication, sub-costal incision, prophylactic nasogastric intubation and abdominal drainage were more common in control group.

**Conclusion:**

Implementation of ERAS protocol is feasible and beneficial for health institutions and patients, without increasing morbidity and mortality.

## RESUMO

**Racional:**

Após a publicação das primeiras recomendações da Sociedade ERAS sobre a cirurgia do cólon, a proposta de redução do estresse cirúrgico, manutenção das funções fisiológicas e recuperação otimizada foi ampliada para outras especialidades cirúrgicas, com pequenas variações.

**Objetivo:**

Analisar a implementação dos protocolos ERAS para cirurgia hepática em um centro terciário.

**Métodos:**

Cinquenta pacientes submetidos à cirurgia hepática eletiva foram avaliados retrospectivamente, utilizando dados de prontuários, de junho de 2014 a agosto de 2016. Após setembro de 2016, 35 pacientes foram prospectivamente avaliados e manejados de acordo com o protocolo ERAS.

**Resultados:**

Não houve diferença de idade, tipos de hepatectomia, cirurgia laparoscópica e complicações pós-operatórias entre os grupos. No grupo ERAS, observou-se redução no jejum pré-operatório e no tempo de internação hospitalar de dois dias (p<0,001). A carga de carboidratos, a incisão em forma de J, a alimentação oral precoce, a prevenção pós-operatória de náuseas e vômitos e a mobilização precoce também foram significativamente relacionadas ao grupo ERAS. Preparo mecânico do cólon, medicação pré-anestésica, incisão subcostal, intubação nasogástrica profilática e drenagem abdominal foram mais comuns no grupo controle.

**Conclusão:**

A implementação do protocolo ERAS é viável e benéfica para instituições de saúde e pacientes, sem aumentar a morbidade e a mortalidade.

## INTRODUCTION

Since the publication of the first enhanced recovery after surgery (ERAS) guidelines regarding colonic resections by Gustafsson et al. in 2012^16^, the proposal of reduction of surgical stress, maintenance of physiological functions and optimized recovery quickly gained the attention of the international medical community. On August 2016, ERAS Society published the official recommendations^26^ for perioperative care for liver surgery, bringing together some experts from high-volume centers all over the world.

Since then, many papers have shown the feasibility and cost-effectiveness of implementation of enhanced recovery pathways in patients undergoing hepatectomies^20^ ^,^ ^34^. In several studies, this multimodal approach was consistently associated with better outcomes, including reduction in the length of hospital stay, postoperative complications and costs^23^ ^,^ ^37^. 

Hepatic surgery represents one of the surgical specialties that most benefits from multidisciplinarity^**1**^ ^**,**^ ^**32**^, but published protocols vary widely between institutions^33^. Despite evidenced-based recommendations available in literature, its application did not follow this progress, mainly due to difficulties in changing paradigms^6^. 

Liver surgery still represents a challenging operation. Despite significant improvements in perioperative management and surgical technique, which led to a reduction in postoperative mortality to less than 5%, major hepatectomies still have a morbidity rate up to 30% in some reports^9^ ^-^ ^11^. This way, the implementation of evidenced-based recommendations in order to optimize perioperative recovery can greatly benefit patients and health providers^3^. 

This is a comparative study that aims to analyze the implementation of ERAS protocols for liver surgery in a tertiary center in Brazil. 

## METHODS

Expertise with implementation of the protocol was initially acquired with colorectal surgery, and it was later expanded to hepatectomies. A database was created with the 23 items proposed by ERAS guidelines, being subsequently filled with the collected data (Table 1). 

**TABLE 1:** Guidelines for enhanced recovery after liver surgery

1.Preadmission counseling
2. Perioperative nutrition
3.Perioperative oral immunonutrition
4.Preoperative fasting and preoperative carbohydrate loading
5.Oral bowel preparation
6.Pre-anesthetic medication
7.Prophylaxis against thromboembolism
8.Perioperative steroids administration
9.Antimicrobial prophylaxis and skin preparation
10. Incision
11. Minimally invasive approach
12. Nasogastric intubation
13. Drainage of the peritoneal cavity
14. Preventing intraoperative hypothermia
15. Postoperative nutrition and early oral intake
16. Postoperative glycemic control
17. Prevention of delayed gastric emptying
18. Stimulation of bowel movement
19. Early mobilization
20. Analgesia
21. Postoperative nausea and vomiting prophylaxis
22. Fluid management
23. Audit

First, it was performed a retrospective evaluation of 50 patients that underwent elective hepatic resections (without biliary anastomosis) at Federal University of Health Sciences of Porto Alegre / Santa Casa Hospital of Porto Alegre, from June 2014 to August 2016, through medical records data (group 1). Patients operated on emergency situations were excluded from analysis. 

Second phase took place between September 2016 and December 2017, and represents the implementation of the protocol itself. Thirty-five patients, managed by the same hepato-pancreato-biliary team, were prospectively evaluated (group 2). 

Nomenclature for liver resections was derived from Brisbane terminology^12^. Major hepatectomies represents removal of three or more Couinaud segments^13^. Definitive diagnosis was obtained from analysis of the pathologic specimen. Complications were classified according to Clavien-Dindo Scale^14^. Preoperative fasting protocol represents light meals ingestion until 6 h before surgery, and no more than 2 h for liquids. Carbohydrate loading with maltodextrin was offered to patients before operation. 

The type of incison, as well as the use of prophylactic abdominal drainage, was performed according to surgeon’s discretion. Normothermia during surgery was maintained using circulating water garments and intravenous warmed solutions. Patients that were at mechanical ventilation after the end of the operation received a nasojejunal tube to guarantee enteral feeding on the first postoperative day (POD1). For the remaining, a liquid oral diet was offered on POD1. 

Postoperative glycemic control was performed with manual injection of insulin according to a pre-established scale. For postoperative analgesia, thoracic epidural, local anesthesia with ropivacain plus intravenous analgesia or intravenous analgesia alone were used. Postoperative nausea and vomiting prophylaxis was accomplished with at least two of the following drugs: ondansentron, metoclopramide or bromopride. All patients that underwent major hepatectomies received a central line at the beginning of the operation, for central venous pressure mensuration during liver transection. The goal was to maintain central venous pressure below 5 mmHg. When necessary, intermittent hepatic pedicle clamping was held (clamping for 15 min, followed by 5 min of declamping).

### Statistical analysis 

Groups were tested for normality by Shapiro-Wilk test. Normal distributions were compared using Student’s t test, and non-normal by Mann-Whitney test. Statistical analysis was performed in the SPSS program version 22.0.0 using the chi-square test of homogeneity, with a level of significance of 5%.

## RESULTS

Table 2 summarizes the participating groups. In total, 85 patients were included in the study, 50 that received the standard care and 35 patients the ERAS program. There were no differences between them regarding age, gender or type of major hepatectomy. There were also no significant differences between the groups in overall and major complications, neither in mortality rate nor pathological findings (Table 2).

**TABLE 2:** Characteristics of the groups.

	ERAS GROUP (35)	CONTROL GROUP (50)	p
Age (years/max-min)	58 (24-78)	60 (22-82)	0.280
Gender (male/female)	16/19	22/28	0.350
Cirrhosis	5 (14.3)	9 (18)	0.080
Major hepatectomy	9 (25.7)	14 (28)	0.430
Hepatectomy technique			
Right Hepatectomy	3 (8.6)	6 (12)	0.093
Left Hepatectomy	5 (14.3)	7 (14)	0.530
Trisectorectomy	1 (2.9)	1 (2)	0.560
Bisegmentectomy	15 (42.9)	21 (42)	0.570
Trisegmentectomy	2 (5.7)	0	0.130
Atipical Resections	9 (25.7)	15 (30)	0.059
Liver pathology			
Colorectal Liver Metastases	13 (37.1)	18 (36)	0.610
Liver Adenoma	5 (14.3)	7 (14)	0.540
Hepatocellular Carcinoma	8 (22.9)	12 (24)	0.645
Gallbladder Neoplasm	1 (2.9)	3 (6)	0.124
Intrahepatic Cholangiocarcinoma	2 (5.7)	2 (4)	0.510
Other	6 (17.1)	8 (16)	0.420

The overall compliance rate before and after the implementation of ERAS protocol was 20% and 65%, respectively. The median postoperative hospital stay was 5 days (2-15) in ERAS group, and 7 (3-22) in control group (p<0.001). A significant number of patients completed the preoperative fasting protocol in ERAS group (70%), and carbohydrate loading with maltodextrin in 80% of them (p<0.001). Oral bowel preparation was omitted in all patients in group 1, and was performed in 24% of patients in group 2 (p=0.001). Similar outcomes were obtained regarding pre-anesthetic medication (p=0.001). Prophylactic nasogastric intubation was held in 62% in group 2, and in only 11.4% in group 1 (p<0.001). Following the same trend, prophylactic abdominal drainage was less common in ERAS group comparing with control (68.6% and 92%, p=0.012).

Regarding the type of incision, the j-shaped one was more prevalent in ERAS group, and bilateral sub-costal in control (29.4% and 69.4% respectively, P<0.001). Thirty-two (91.4%) of patients started enteral feeding on POD1 in ERAS group, being 82% by oral route. This proportion was significant higher than in group 2 (50%, p<0.001). Similarly, 82.9% and 88.6% of patients started postoperative early mobilization and proper postoperative nausea and vomiting prophylaxis in ERAS group (p=0.001). Of note, 100% of patients in ERAS group underwent systematic audit; this data was missing in control group. Table 3 summarizes the main outcomes.

**TABLE 3:** Main outcomes after ERAS implementation.

VARIABLES	ERAS (n=35)	CONTROL (n=50)	p
Length of hospital stay, median (min-max)	5 (2-15)	7 (3-22)	<0,001
Immunonutrition	0 (0,0)	1 (2,0)	0,928
Carbohydrate loading	28 (80)	0 (0,0)	<0,001
Oral bowel preparation	0 (0,0)	12 (24,0)	0,001
Pre-anesthetic medication	0 (0,0)	12 (24,0)	0,001
Anti-thrombotic prophylaxis	34 (97,1)	48 (96,0)	>0,999
Perioperative steroid administration	19 (54,3)	8 (40,0)	0,460
Antimicrobial prophylaxis	35 (100,0)	48 (96,0)	0,510
Incision			0,001
J-shaped	10 (29,4)	3 (6,1)	
Bilateral subcostal	10 (29,4)	34 (69,4)	
Laparoscopy	14 (40,0)	12 (24,0)	0,181
Nasogastric intubation	4 (11,4)	31 (62,0)	<0,001
Prophylactic abdominal drainage	24 (68,6)	46 (92,0)	0,012
Preventing intraoperative hypothermia	33 (94,3)	36 (94,7)	>0,999
Postoperative nutrition POD1	32 (91,4)	25 (50,0)	<0,001
Postoperative glycemic control	24 (68,6)	29 (58,0)	0,446
Omental flap	2 (5,7)	0 (0,0)	0,167
Stimulation of bowel movement	6 (17,1)	6 (12,0)	0,540
Early mobilization	29 (82,9)	19 (38,0)	<0,001
Analgesia			
Intravenous	17 (48,6)	19 (38,0)	0,455
Epidural	18 (51,4)	31 (62,0)	0,455
Local	14 (40,0)	11 (22,0)	0,121
Postoperative nausea and vomiting prophylaxis	31 (88,6)	27 (54,0)	0,001
Fluid management	27 (77,1)	17 (81,0)	>0,999
Audit	35 (100,0)	-	-
Overall Complications	8 (22.9)	12 (24)	0.878
Dindo-Clavien ≥ 3	4 (11.4)	7 (14)	0.230
Mortality	0	1 (2)	0.720

## DISCUSSION

Enhanced recovery programs (ERP), together with the development of minimally invasive approach and strategies to improve liver hypertrophy represent the greatest advances in hepatic surgery in the last decade^10^ ^,^ ^26^ ^,^ ^35^. ERAS protocols are the most recent of them, bringing the concept of a multimodal pathway to achieve better results^26^. 

Probably, the most reproduced outcome in papers comparing ERAS guidelines with traditional care is the length of hospital stay (LOS). Liang et al.^24^, evaluating patients that underwent laparoscopic hepatectomies according to ERAS protocols in China, showed a decrease in the median postoperative hospital stay in ERAS group of approximately three days. Similar conclusion was reported in a meta-analysis published by Li et al.^23^ in 2017, analyzing 254 patients treated according to ERAS guidelines. They verified that the postoperative recovery time and length of hospital stay were significantly better in this group. These two studies show that the benefit is not only related to laparoscopy itself, but also to the compilation of evidenced-based steps that work together to optimize perioperative recovery. In our work, we reduced the LOS in two days, even with the same rate of laparoscopic hepatectomies in both groups. This result is in line with recent reports^10^.

In our study, a significant number of patients completed the fasting protocol, with light meals intake until 6 h before surgery, and carbohydrate loading with maltodextrin 2 h before operation. These measures not only give comfort and reduce anxiety in preoperative hours, but are also related to a reduction in catabolism and insulin resistance in some papers^2^ ^,^ ^3^ ^,^ ^14^. Of note, we did not verified and increase in perioperative complications (like aspiration during anesthesia or postoperative pneumonia) following these recommendations. The same line of reasoning can be made for oral bowel preparation^17^, pre-anesthetic medication^25^ and prophylactic nasogastric intubation^18^ ^,^ ^27^ ^,^ ^29^: its omission could be done safely. 

Prophylactic abdominal drainage remains an area of uncertainty after liver surgery. Since the first evidenced-based publications regarding the use of prophylactic drains after abdominal operations^5^ ^,^ ^30^, the debate about its real benefit in preventing postoperative complications after hepatic resections came to light. 

A recent study by Brauer et al.^7^, analyzing databases of several American institutions, showed that drainage of the surgical site after hepatectomies did not improve the rate of diagnosis of major biliary leaks, in addition to increase the number of interventions, the LOS and 30-day readmissions. On the other hand, Kyoden et al.^30^, in 2010, questioned the design of previous studies that disfavored the routine use of drains, as well as its management in the postoperative period. Evaluating the value of prophylactic drainage in 1269 consecutive hepatectomies performed at the University of Tokyo, they concluded that prophylactic drainage was effective in reducing the frequency of subphrenic collections and biliary fistulas in a large number of patients.

Enhanced recovery pathways, in general, discourage the routine use of drains, as there is some evidence that a no-drain policy is safe and feasible after uncomplicated hepatectomies^39^. In our cohort, there was a significance reduction in the placement of abdominal drains in ERAS group, without increasing complications like infected collections, hemorrhage, percutaneous drainage or reoperations.

Minimally invasive liver surgery (MILS) still represents a challenge operation even for experienced surgeons^**28**^. However, after the publication of two consensus giving recommendations about laparoscopic liver resections, its use has grown and spread throughout the world, mainly because of the benefits related to the method, as less wound complications and postoperative pain, early mobilization and a decrease in LOS^8^ ^,^ ^36^. Despite this, it requires specific material for its adequate fulfillment, which is not available in the Brazilian public health system. Thus, in our series, even with and increase in MILS in ERAS group, the 40% report rate is far behind from our expectations, especially when we have 68% of patients that underwent bisegmentectomies or atypical resections. 

Therefore, many hepatic resections in our series were performed by laparotomy. Although there is not a strong recommendation regarding the better type of incision, in the intervention group a greater number of patients were operated on by a J-shaped incision, differently from control group in which a bilateral sub-costal was more prevalent (p=0.001). Saving the left rectus muscle by a J-shaped incision contributes to a decrease in postoperative pain and better ventilation, with the same exposure of the operative field^9^. 

Early enteral intake on POD1 could be resumed in the vast majority of our patients (91%), being oral route in 82% of them. Lee et al.^22^, in Korea, showed that for patients undergoing liver resection, the early enteral diet (on the first postoperative day) resulted in a decrease in the LOS and faster return of gastrointestinal tract function. Yan et al.^40^, in a meta-analysis, reported similar benefits with early enteral feeding in surgical patients with gastrointestinal neoplasms. The enteral diet, compared to parenteral, reduced pulmonary and operative wound infections, and also the occurrence of anastomotic fistulas. We believe that the enteral diet should always be attempted; besides of previous benefits, it comforts patients without increasing morbidity, as the majority of them will be able to tolerate diet earlier in the postoperative period. 

Early mobilization and adequate postoperative nausea and vomiting prophylaxis were the other steps from ERAS guidelines that could be accomplished in more than 80% of patients in the intervention group. The former requires and intensive participation of physiotherapist and nursing personnel. Yip et al.^36^ demonstrated that sitting out of the bed in the 1^st^ PO and walking in the 3^rd^ PO were factors related to compliance of ERAS protocol in their institution, which includes allowing them to be discharged within six days. We share the same opinion, and using a multidisciplinary approach we look for guaranteeing general well-being and activities of daily life as soon as possible. Regarding PONV prophylaxis, using at least two medications with different mechanisms of action represents the most recommended model for prophylaxis, and has been proposed for high-risk patients^15^. This strategy enables adequate enteral feeding and, ultimately, early discharge. 

Interestingly, a 100% of patients underwent systematic audit in our work group, which increases the power of this study. A systematic review by Cochrane has shown that audit and feedback may represent useful strategies for improving compliance with established measures^19^. In addition, they reflect the results of an Institution more accurately, serving as a method to compare professionals with their peers, always seeking to achieve better outcomes. 

To our knowledge, this study represents the first Brazilian experience with ERAS regarding hepatic resections, but it has some limitations. The retrospective analysis in the control group confers risks for the occurrence of selection biases and loss of data. This fact was minimized by recovering the last 50 patients operated on by our team for comparison with ERAS group, in the same way as guided by the ERAS Society. In addition, we had a small number of patients for analysis in the intervention group, which, theoretically, could limit its reproducibility in other settings. However, even with a small sample, statistical analysis showed significance at several points of the protocol, making it possible to accomplish the initial objective of the study.

## CONCLUSION

Implementation of enhanced recovery protocols in liver surgery is feasible and beneficial for patients and care providers and reduced the length of hospital stay in two days. This represents a positive impact on perioperative care of patients undergoing hepatectomies, possibly saving costs without increasing morbidity and mortality. Looking for a better compliance to the established recommendations of ERAS Society may result in even better outcomes. 
